# Society Meetings

**Published:** 1906-10

**Authors:** 


### SOCIETY MEETINGS.

The sixth annual conference of the Sanitary Officers of the State of New York will be held at Syracuse October 24-26, 1906. The conference will be addressed by Dr. Eugene H. Porter, State Commissioner of Health, Dr. Samuel Dixon, Commissioner of Health of the State of Pennsylvania, and Col. Charles W. Fuller, chairman of the Sewage Commission of the State of New Jersey. The mayor of Syracuse, Hon. Alan C. Forbes; the president of the chamber of commerce, Hon. Giles H. Stilwell; and the chancellor of Syracuse University, Hon. James R. Day, will deliver addresses of welcome. Eight sessions are scheduled during which papers will be read and discussed relating to public health problems, and exhibitions and demonstrations made concerning sanitary topics.

The Buffalo Academy of Medicine held meetings during September as follows:

*Section of Medicine.*—Tuesday, Sept. 18, 1906, at 8:30 P. M. Program: (a) Demonstration of Prof. Irving Fisher's apparatus for the mechanic estimation of dietaries, A. L. Benedict. (b) The essential action of some common drugs, Eli H. Long. (c) Cysts of the spinal cord, William C. Krauss.

A Stated Meeting of the Academy was held Tuesday, Sept. 25th, 1906, at 8:30 P. M. Meeting of the council at 8:15 sharp. The program of the evening was furnished by the Section of Obstetrics and Gynecology, as follows: (a) Malpositions of the uterus, John V. Woodruff. (b) Principal cause for abnormal head presentation, Thomas G. Allen. A collation was served at the close of the meeting.

The Medical Association of Central New York will hold its Thirty-Ninth Annual Meeting at Syracuse, Tuesday, October 16, 1906, under the presidency of Dr. D. M. Totman of that city. The secretary is Dr. C. A. Greenleaf, of Canoga. The program had not been issued when this notice was written.

The Medical Society of the County of Erie will hold an adjourned meeting at the University Club, Buffalo, Monday evening October 8, 1906, at 8:15 o'clock, "for the purpose of receiving and acting upon the report of the counsel of the society as to whether the recently proposed by-laws conflict with the statutes of the State of New York, and such other matters as may properly come before the meeting." It were well if this society would at once conform to the new order of things resultant from the amalgamation of the two state medical bodies, and their constituent organisations, without further speciousness or quiddity.

